# Trauma related psychiatric disorders and their correlates in a clinical sample: A cross-sectional study in trauma affected patients visiting a psychiatric clinic in Nepal

**DOI:** 10.1371/journal.pone.0234203

**Published:** 2020-06-15

**Authors:** Rishav Koirala, Erik Ganesh Iyer Søegaard, Saroj Prasad Ojha, Edvard Hauff, Suraj B. Thapa

**Affiliations:** 1 Division of Mental Health and Addiction, Institute of Clinical Medicine, University of Oslo, Oslo, Norway; 2 Brain and Neuroscience Center, Kathmandu, Nepal; 3 Division of Mental Health and Addiction, Oslo University Hospital, Oslo, Norway; 4 Department of Psychiatry and Mental Health, Institute of Medicine, Tribhuvan University Teaching Hospital, Kathmandu, Nepal; Yale University, UNITED STATES

## Abstract

**Background:**

Nepal, like many other low-income countries, has a great burden of mental health issues but few resources to meet them. In addition, Nepal has endured several traumatic events in recent decades but the impact on mental health has not been studied in clinical settings. This study explores trauma-related psychiatric disorders and their correlates.

**Methods:**

100 patients with a history of trauma who visited the outpatient psychiatry clinic at a University hospital in Kathmandu were assessed. The Composite International Diagnostic Interview 2.1 (CIDI) was used to evaluate lifetime and current depressive disorder, generalized anxiety disorder (GAD) and lifetime post-traumatic stress disorder (PTSD). Current PTSD was evaluated using PSTD Checklist—Civilian Version (PCL-C).

**Results:**

The median number of lifetime traumatic events was two. Natural disaster was the most common trauma type (84%) compared to other types of trauma. Rape was reported as the most traumatizing. Current PTSD was found in 15%, depression in 33% and GAD in 38% of the patients. The lifetime rates were PTSD 83%, depression 45% and GAD 40%. There was high comorbidity between the disorders. The 31 to 45 years age group, above high school education level and trauma types other than earthquake were independently associated with current PTSD. Marital status and upper socioeconomic status (SES) compared to upper-middle SES were independently associated with lifetime PTSD. Both lifetime and current depression rates were independently associated with the upper SES compared to upper-middle SES. Place of living, education above high school and lower-middle SES were significantly associated with lifetime and current GAD.

**Conclusion:**

PTSD, depression and GAD were prevalent in a trauma exposed patient population visiting a psychiatric clinic in Nepal. High rates of comorbidities and several risk factors were identified. Our findings highlight the need for addressing trauma related disorders in clinical settings in developing countries.

## Introduction

Low-income countries have a heavier burden of mental illness than high-income countries, with a higher prevalence of certain disorders and lower access to treatment [1, 2]. It has been seen that poverty and mental health have a bidirectional relationship hence creating a vicious cycle [3, 4]. Less than 5% of the total population suffering from mental health issues receive proper care in low-income countries [1, 5, 6]. Along with that, the prevalence of trauma-related illness is even higher in countries emerging from conflict or natural disaster [7]. Exposure to trauma can lead to several types of mental illness, mainly, depression, anxiety disorders, post-traumatic stress disorder (PTSD) and adjustment disorders [8, 9].

Nepal is a country located in South Asia with a population of 29.3 million [10]. Like in other low-income countries, the mental health system in Nepal is not well developed. The major challenges faced in the development of mental health in Nepal are a lack of human resources [11], stigma towards mental health issues [12, 13] and poor allocation of health funds into mental health [14].

A devastating earthquake of 7.8 moment magnitude hit Nepal in April 2015 killing 8,669 people and damaging over 200,000 buildings [15]. It occurred at the moment when Nepal was recovering from a decade long (1996–2006) period of civil unrest which cost over 15,000 lives and caused many to flee from their homes to save their lives [16]. After the earthquake, the need to address trauma and stress related disorders was more highlighted; previously, this had not a priority in Nepal [17].

There has been no nationwide epidemiological study of the prevalence of mental disorders in Nepal. Very few studies for PTSD in the general population have been done in Nepal. One of the major studies has shown the prevalence of PTSD to be 9.6% in the general population [18], which is higher than was found by the World Mental Health Survey (WMHS): the WMHS found lifetime prevalence of PTSD to be 3.9% in the total population and 5.6% in trauma exposed population [19]. Similarly, lifetime depression in general populations has been seen to range from 6% to 12% globally [20] and 11.7% in Nepal [21]. The prevalence of current anxiety disorder in general population of Nepal is 22.7% [21], which is higher than the WMHS 12-month prevalence rate of 9.8% [5]. Previous community studies in trauma-affected populations in Nepal have reported prevalence rates of PTSD ranging from 14% to 59.7% [22–24]. In the same population, rates of depression and anxiety have been identified from 7% to 81%, and 6.3% to 85.5%, respectively [18, 24, 25]. Similarly, in the global context, very wide prevalence ranges have been identified for both PTSD (0% to 99%) and depression (3% - 85.5%) in trauma-exposed populations [26]. The latest community level studies on earthquake affected victims of Nepal have reported the rates of PTSD as 27% in the 14^th^ month after the earthquake [27] and 24% in the 20^th^ month after the earthquake [28]. Despite the high prevalence of PTSD, diagnosing trauma related disorders in clinical practice is not common in Nepal. Moreover, there has been the preformed idea among many mental health professionals that “PTSD does not exist in Nepal” [29]. To our knowledge, there has been no major study of trauma related disorders in hospital settings in Nepal. It has been established that neglecting and not treating PTSD can make it chronic, more severe, and highly incapacitating [30]. Furthermore, inquiring about traumatic life events as well as assessing for PTSD in the clinical setting is also less commonly practiced in many parts of the world [31].

Trauma is unrecognized; trauma disorders are underdiagnosed and undertreated in clinics in Nepal. Hence we chose a special setting, the psychiatric outpatient department (OPD) in a tertiary hospital, and a special group of people, trauma-exposed participants, where we expected to find high rates of affected patients seeking help. Through this research we hope to bring into notice among the mental health workers working in Nepal, the high prevalence of trauma affected illness in this special group of people seeking help. This particular study examines the rates of depression, GAD, and PTSD in these patients. Further, we examined the characteristics of trauma incurred and explored associations of background variables including trauma variables with psychiatric disorders.

## Materials and method

### Study population

This is a cross-sectional study on patients attending the psychiatric OPD of the Tribhuvan University Teaching Hospital, Institute of Medicine (TUTH, IOM), Nepal. This is one of the largest hospitals of Nepal, which has been providing mental health services to people from all over the country since 1986 [32]. This research is part of the Study of Health Outcomes of Trauma (SHOT) being conducted simultaneously in Norway and Nepal, a broad study in which biological parameters such as genetics and biochemical markers (cytokines) are also included.

The participation inclusion criterion was the experience of at least one trauma as defined by International Classification of Diseases version 10 (ICD-10) in the PTSD section [33] at least one month before the interview and an age between 18 and 60 years. Patients with serious medical, neurological or mental disorders were excluded.

### Interview procedure

All new patients attending the psychiatry OPD of TUTH were asked if they had experienced trauma. If so, they were informed about the research project and allowed to participate by signing a written consent. For illiterate patients, the consent paper was read to them and the accompanying persons were asked to sign the consent on their behalf.

The first author, a psychiatrist, collected the data under supervision. All patients were interviewed for an average of 2 hours. On the next day, the laboratory examinations were completed. In the main study, all the patients who had trauma history consented to participate in the study. Two of the 102 patients interviewed were excluded from the final analysis, as they were older than 60 years. The data collection was done from 1^st^ April 2017 to 14^th^ August 2018.

### Measures and instruments

#### Socio-demographic questionnaire.

A questionnaire was created to assess sociodemographic characteristics, which included six variables: gender, age, place of living, education, marital status and religion. Age consisted of three categories (in years): 18–30, 31–45, and 46–60. Marital status was classified into two groups: married and single (including unmarried, divorced or widower). Place of living was categorized either as urban or rural. Education was classified into three categories: illiterate, up to high school and above high school. As the majority of population of Nepal identify as Hindu, religion was categorized into Hindu and other (i.e., any other religion). The Kuppuswami socioeconomic scale was used to measure socioeconomic status of the patients. This is a widely used scale to evaluate the SES of the South Asian populations [34], which has been modified to fit the Nepalese context [35]. We combined the last two categories of this scale (upper-lower and lower SES) as very few participants fell into each category.

#### WHO World Mental Health Composite International Diagnostic Interview (WMH-CIDI) version 2.1.

The CIDI is a comprehensive, standardized diagnostic interview designed for assessing mental disorders according to the definitions of the Diagnostic Criteria for Research of ICD-10 [36]. CIDI version 2.1 has been translated and validated in Nepali [37]. Section K includes a list of ten possible traumatic events. It gives an overview of the traumatic events that one may have gone through in his/her lifetime.

#### PTSD symptom checklist civilian version (PCL-C).

This instrument is used for measuring the load of PTSD symptoms [38], consisting of 17 items assessed on self-rating scale for PTSD. The checklist of symptoms was developed by the Behavioral Science Branch of American PTSD research center in 1994. It uses DSM-IV criteria for evaluating the experiences of general people after trauma in normal civilian life. Among the 17 items of PCL-C, 12 of them correspond with criteria of PTSD as defined by ICD-10. Individuals rate each item on a Likert scale from 1 (not at all) to 5 (extremely) to indicate the degree of subjective symptoms over the past month. PCL-C was translated and validated in Nepali [22]. We used ICD-10 criteria to diagnose current PTSD.

### Statistical analysis

The statistical program SPSS version 25 was used for the statistical analysis [39]. Chi-square test was used to compare the categorical variables with psychiatric disorders (both current and lifetime), which is equivalent to conducting unadjusted logistic regression models. We used Fisher’s exact test values for variables that had a count of less than 5 in a cell. Clinically significant variables with Variance Inflation Factor <2 were included in the final model. Multivariate logistic regression analyses were run with multiple predictors to identify independent factors associated with psychiatric disorders (both current and lifetime). Age, gender, marital status, place of living, religion, education, trauma frequency and type were used as independent variables in the final models. The alpha level was set at p<0.05.

### Ethical consideration

The study was approved by the Institutional Review Board of IOM, reference no 278(6-11-E) 073/074; Nepal Health Research Council (NHRC), reference no 801 and Regional Ethical Committee of South-Eastern Norway (REK Sør-Øst), reference no. REK 2015/2081.

## Results

### Sociodemographic characteristics and predictors of trauma exposure

A total of 100 patients were included in the study. For current PTSD, one participant was discarded due to incomplete entry of the PCL-C. The mean age of the participants was 33.3 years (range 18–60). Forty-eight percent were male and fifty-two percent were female. Most of the participants experienced trauma when they were 20 to 29 years old (34%), followed by 30 to 39 years (26%). Over half (56%) of the patients had basic primary education (1–10 years). The majority of the study subjects identified as Hindu (82%). Participants were evenly distributed between living in a rural (49%) and urban area (51%). Most of the patients belonged to the middle class SES (80%) (Table 1).

**Table 1:** Sociodemographic profile.

Gender	Percentage
Male	48
Female	52
**Age (years)**	
18–30	43
31–45	42
46–60	15
**Education**	
Illiterate	17
Upto high school	56
Above high school	27
**Religion**	
Hindu	82
Others	18
**Place of living**	
Rural	49
Urban	51
**Socioeconomic status**	
Upper class	12
Upper-middle class	46
Lower-middle class	34
Lower-class	8
**Marital Status**	
Married	71
Single	29
**Age at the exposure of trauma**	
10 to 19 years	16
20 to 29 years	34
30 to 39 years	26
40 to 49 years	18
50 to 59 years	5
60 years	1

### Frequency and distribution of trauma types

From the Trauma Checklist CIDI Section K22, the mean number of reported lifetime trauma events was 1.89 and the median was 2. Natural disaster (earthquake in all cases) was the most frequent type of trauma (Table 2). Life-threatening accidents were the second most common type of trauma.

**Table 2:** Characteristics of trauma.

Number of traumatic events	
Median	2
Range	1–5
**Types of trauma (both primary and secondary)**	**Percentage**
Natural disaster	84
Life threatening accident	29
Witness murder/ rape/ grievous injury	19
Seriously physically attacked or assaulted	16
Kidnapping/ threatened with weapon	11
Sexual assault (other than rape)	8
Close one suffering from stressful event	7
War related trauma	6
Rape	5
Any other extremely stressful events	3
Torture/ victim of terrorist	0
**Most traumatizing event (Primary trauma)**	Number of participants (%*)
Natural disaster (earthquake)	57 (67.8%)
Life threatening accident	11 (37%)
Kidnapping/ threatened with weapon	7 (63.6%)
Witness murder/rape/grievous injury	6 (31.6%)
Rape	5 (100%)
Sexual assault (other than rape)	5 (62.5%)
Seriously physically attacked or assaulted	4 (25%)
Close one suffering from stressful event	3 (42.9%)
Any other extremely stressful events	1 (33.3%)
War related trauma	1 (17%)
Torture/ victim of terrorist	0 (0%)

* relative in-group percentage.

All of the five victims of rape reported it as being the worst trauma in their lives, even though all five had experienced other traumas as well. Among the five who experienced sexual assault, 63% regarded it as their worst trauma.

### Trauma related psychiatric disorders

Table 3 shows the rates of current and lifetime PTSD, depression and GAD with certain socio-demographic variables.

**Table 3:** Rates of psychiatric disorders according to sociodemographic variables.

	Lifetime			Current		
	PTSD*	Depression	GAD	PTSD	Depression	GAD
**Gender**						
Male (48)	40 (83.3)	20 (41.7)	15 (31.3)	7 (14.6)	16 (33.3)	14 (29.2)
Female (52)	43 (82.7)	25 (48.1)	25 (48.1)	9 (17.6)	17 (32.7)	24 (46.2)
**Age groups**						
18–30 years (43)	35 (81.4)	24 (55.8)	15 (34.9)	10 (23.8)	16 (37.2)	14 (32.6)
31–45 years (42)	37 (88.1)	15 (35.7)	19 (45.2)	3 (7.1)	12 (28.6)	19 (45.2)
46–60 years (15)	11 (73.3)	6 (40.0)	6 (40)	3 (20.0)	5 (33.3)	5 (33.3)
**Education**						
Illiterate (17)	13 (76.5)	8 (47.1)	8 (47.1)	2 (11.8)	5 (29.4)	7 (41.2)
Upto high school (56)	47 (83.9)	21 (37.5)	18 (32.1)	7 (12.7)	19 (56.0)	17 (30.4)
Above high school (27)	23 (85.2)	16 (59.3)	14 (51.9)	7 (25.9)	9 (27)	14 (51.9)
**Religion**						
Hindu (82)	70 (85.4)	39 (47.6)	32 (39.0)	13 (16)	27 (32.9)	30 (36.6)
Others (18)	13 (70.2)	6 (45)	8 (44.4)	3 (16.7)	6 (33.3)	8 (44.4)
**Place of living**						
Rural (49)	41 (83.7)	23 (46.9)	15 (30.6)	7 (14.3)	15 (30.6)	14 (28.6)
Urban (51)	42 (82.4)	22 (43.1)	25 (49.0)	9 (18.0)	18 (35.3)	24 (47.1)
**SES**						
Upper class (12)	7 (58.3)	4 (33.3)	4 (33.3)	1 (8.3)	1 (8.3)	4 (33.3)
Upper-middle class (46)	42 (91.3)	21 (45.7)	17 (37.0)	9 (19.6)	16 (34.8)	15 (32.6)
Lower-middle class (34)	28 (82.4)	17 (50)	16 (47.1)	5 (15.2)	15 (44.1)	16 (47.1)
Lower class (8)	6 (75)	3 (37.5)	3 (37.5)	1 (12.5)	1 (12.5)	3 (37.5)
**Marital Status**						
Married (79)	68 (86.1)	36 (42.9)	32 (38.1)	13 (15)	24 (42.9)	31 (39.2)
Single (21) (divorce/unmarried/ widower)	15 (71.4)	9 (45.6)	8 (40.5)	3 (16.5)	9 (30.4)	7 (33.3)

^*^ The relative in-group percentage of participants in each category is written inside the bracket behind the actual values.

We assessed bivariate associations between sociodemographic variables and psychiatric disorders, using Chi-square test, which is equivalent to running unadjusted bivariate logistic regression models for these independent variables. Lifetime PTSD diagnosis had a significant association with SES (p = 0.04). Similarly, lifetime GAD also had near-significant association with experiencing the earthquake as a traumatic event (p = 0.053). There were no other statistically significant associations between other psychiatric illnesses and socio-demographic characteristics as shown in Table 3.

### PTSD and its comorbidity with other mental illnesses

The comorbidity of current PTSD with current depression and current GAD were 62.5% and 43%, respectively. Similarly, the comorbidity of lifetime PTSD with lifetime depression was 49% and with lifetime GAD was 43%.

### Factors independently associated with psychiatric disorders

For our multivariate model, we selected variables that were significant and near- significant in our bivariate analyses (Chi-square tests) or shown in previous studies to have associations with psychiatric disorders. We ran logistic regression analyses between these variables as explanatory variables using psychiatric disorders as dependent variables. The 31–45 years age group, above high school education level and trauma types other than earthquake were independently associated with current PTSD (Table 4). The odds ratio of current PTSD was 0.12 in the 31–45 years age group compared to the 18–30 years age group. Similarly, people with education above high school were 7.27 times more likely to have current PTSD than those with below high school of education. Marital status and upper SES in comparison to upper-middle SES were independently associated with lifetime PTSD. Single participants were .132 times less likely to have lifetime PTSD in comparison to the married ones. Similarly, the odds of lifetime PTSD was 0.14 for upper SES compared to upper-middle class SES.

**Table 4:** Factors independently associated with psychiatric disorders among patients with trauma history.

	Lifetime						Current					
	PTSD		Depression		GAD		PTSD		Depression		GAD	
**Variables**	OR (95% CI)	p value	OR (95% CI)	p value	OR (95% CI)	p value	OR 95% CI	p value	OR 95% CI	p value	OR 95% CI	p value
Age (18–30) (ref)	1		1		1		1		1		1	
Age (31–45)	1.75 (.39–7.52)	.481	.364 (.131–1.01)	.053	1.90 (.655–5.54)	.237	.116 (.020 –.657)	**.015**	.618 (.219–1.74)	.363	2.10 (.709–6.24)	.180
Age (46–60)	.824 (.112–6.07)	.849	.700 (.132–3.71)	.675	2.76 (.497–15.3)	.246	1.78 (.187–17.0)	.615	1.47 (.248–8.69)	.672	2.47 (.417–14.7)	.319
Gender (male) (ref)	1		1		1		1		1		1	
Gender (female)	1.01 (.266–3.9)	.978	1.11 (.420–2.94)	.832	1.89 (.680–5.24)	.223	1.14 (.286–4.54)	.854	.827 (.301–2.27)	.713	2.14 (.744–6.18)	.158
Marital status (married) (ref)	1		1		1		1		1		1	
Marital Status (single, widowed or separated)	.132 (.023 - .771)	**.025**	.434 (.12–1.57)	.202	1.51 (.406–5.64)	.537	.171 (.029–1.01)	.052	1.217 (.35–4.22)	.758	1.00 (.258–3.88)	.999
Religion (Hindu) (ref)	1		1		1		1		1		1	
Religion (other than hindu)	. 441 (.060–3.22)	.420	.657 (.145–2.97)	.586	.684 (157–2.98)	.614	3.153 (.409–24.3)	.270	1.6 (.366–7.05)	.531	1.01 (.225–4.51)	.992
Place of living (rural) (ref)	1		1		1		1		1		1	
Place of living (urban)	1.15 (.267–4.94)	.853	1.13 (.410–3.09)	.819	3.48 (1.11–10.9)	**.032**	1.58 (.372–6.74)	.535	1.80 (.630–5.16)	.272	3.62 (1.13–11.6)	**.030**
Education (up to high school) (ref)	1		1		1		1		1		1	
Education (illiterate)	.369 (.042–3.22)	.367	1.35 (.265–6.92)	.716	1.59 (.288–8.77)	.594	.248 (.024–2.60)	.245	.884 (.158–4.95)	.888	1.13 (.200–6.40)	.888
Education (above high school)	5.59 (.494–63.1)	.164	6.88 (1.78–26.6)	.**005**	5.88 (1.52–22.8)	**.010**	7.27 (1.44–36.8)	**.017**	2.71 (.769–9.57)	.121	7.47 (1.83–30.5)	**.005**
SES^*^ (upper-middle class) (ref)	1		1		1		1		1		1	
SES (upper class)	.014 (.001– .209)	**.002**	.15 (.024 - .93)	**.042**	.309 (.055–1.72)	.180	.089 (.006–1.31)	.078	.085 (.008 - .900)	**.041**	.313 (.055 - .178)	.190
SES (lower-middle class)	.399 (.078–2.04)	.270	11.96 (1.51–94.7)	.285	4.04 (1.18–13.8)	.**026**	1.49 (.321–6.95)	.609	2.56 (.848–7.71)	.096	5.99 (1.64–21.9)	**.007**
SES (lower class)	1.15 (.091–14.7)	.912	1.20 (.136–10.5)	.871	2.07 (.248–17.3)	.501	.423 (.022–8.24)	.570	.242 (.020–2.86)	.261	2.86 (.335–24.5)	.336
Trauma frequency (less than 2 type) (ref)	1		1		1		1		1		1	
Trauma frequency (more than 2 type)	.862 (.196–3.79)	.845	2.64 (.840–8.32)	.096	.578 (.166–2.02)	.390	.575 (.107–3.08)	.518	2.02 (.633–6.42)	.236	.604 (.170–2.15)	.436
Type of trauma (earthquake) (ref)	1		1		1		1		1		1	
Type of trauma (other than earthquake)	1.72 (.427–6.94)	.446	1.61 (.599–4.33)	.345	.355 (.122–1.03)	.057	5.33 (1.24–22.8)	**.024**	.926 (.331–2.59)	.884	.468 (.158–1.39)	.171

OR: Odds Ratio; CI: Confidence Interval; ref: reference; SES: Socio-economic status.

Both lifetime and current depression rates were independently associated with the upper SES in comparison to upper-middle class SES. Participants with education above high school had a probability of 6.88 times than those with education up to high school of having lifetime depression. No other test variables showed an association with lifetime and current depression. Place of living, education above high school and lower-middle class of SES were statistically significantly associated with lifetime and current GAD.

## Discussion

In our study, when the characteristics of trauma were explored, the median number of trauma types experienced in life was 2. Natural disaster was the most common trauma type and it was also regarded as the most traumatizing event. More than half of the patients reported about their trauma only after being specifically asked about it. This finding may implicate a lack of understanding of the impact of trauma on mental health, especially in the setting of a developing country. The link between trauma and its effects on mental health is poorly understood in many parts of the world as well [40]. In a large international study, the mean number of trauma types was 2.9 among those with any trauma and 2.0 in the general population [41]. Due to the recent earthquake in Nepal, many participants (87%) had experience of natural disaster as trauma. One possible explanation for the high percentage of natural disasters as the most traumatizing event maybe because majority in this group was only exposed to natural disaster and had not experienced other forms of trauma. The rates of participants who were victims of sexual abuse/rape may well be higher than stated (13%), as many do not disclose these types of experiences in their first visit. These patients regarded sexual trauma/ rape as the most traumatizing event: 100% for rape and 62.5% for sexual abuse.

We have found a high lifetime PTSD rate (83%) among our study subjects in comparison to the general population. Our results correspond well with findings from a similar study in Norway which found PTSD rate around 89% in the trauma affected patients seeking help in a psychiatric clinic [42]. Studies done in hospitals or outpatient settings have found a wide range of prevalence of PTSD, ranging from 11.2% to 69% [31, 43, 44]. Similar rates have been seen in conflict-affected settings in Nepal and internationally [24, 26]. We should keep in mind that these findings represent a special group of population and do not represent the general population where the prevalence rate is significantly lower, 3.9% in total population and 5.6% in trauma exposed population [19].In Nepal, there seems to be a high threshold for seeking help for psychological problems. This may be due to stigma and a lack of available services. This may be one explanation for the high rates of diagnoses in people visiting psychiatric clinics [45, 46].

Current PTSD rates were comparable to findings from other parts of the world [7]. A recent systematic review and meta-analysis conducted by the WHO as well as other studies in the trauma-affected population in both Nepal and worldwide also support this finding [7, 22, 47]. Despite this, many mental health experts in Nepal very rarely diagnose PTSD. PTSD may be underdiagnosed by mental health professionals due to several reasons. There has been a preconception among many mental health professionals that PTSD does not exist in Nepal [29]. Since PTSD is highly comorbid with other common illnesses such as depression and GAD [48–50], it might be missed during evaluation. Patients not acknowledging the impact of trauma may also lead to underreporting of the trauma related illness [40]. Nepali culture does not have a construct that parallels a PTSD diagnosis. Thus both clinicians and patients themselves may be blind to the possibility of having PTSD. This trend is found in other parts of the world as well [31, 51].

High rates of lifetime depression (45%) and current depression (33%) were seen in this special population, similar to previous studies in Nepal and in other countries [48, 50]. Though these scores are higher than those found by the WHO after a humanitarian crisis/ trauma exposure, they are similar to findings from a recent meta-analysis [26, 52]. In another meta-analysis, half of participants with current PTSD had depression [53]. We should keep in mind that these rates are higher than that shown in general population. In a study done in general population in Nepal depression was seen to be 4.2% [21]. This variation of prevalence of mental illness in different regions as evaluated in different studies has been attributed to different methodologies (e.g., operational definitions of terms), different types of trauma and variations in support received [26, 41]. More than one-third of the patients had lifetime GAD and 95% still had symptoms. In a population study done among refugees in Nepal, only 6% of the participants had GAD whereas 43% of them had PTSD [25]. In a study done in general population of Nepal, GAD was observed in 16.1% [21]. Closely similar rates of lifetime and current GAD can be attributed to the persistent nature of the illness, and highlights the fact that there is a higher burden of disease in this group of patients. Hence, clinicians should be aware of the trajectories of the impact of trauma and manage accordingly.

The World Mental Health survey, one of the largest studies of its kind, showed that correlates of PTSD differs significantly between cultures and countries [19, 54].

In our study, being single was a protective factor against lifetime PTSD. Marriage has been shown to be a protective factor in some studies [55, 56] while another study showed it has a negative role [57]. In Nepal there is significantly increased responsibility associated with marriage as arranged marriages are common and some marriages are forced [58], which will further increase the risk of mental illness. Our study showed that participants from the upper SES category had lower chances of having PTSD than the upper-middle class, which is supported by previous studies [56]. In Nepal the quality of healthcare provided by the government is not adequate. People mainly rely on private hospitals for better healthcare services. Hence, the upper SES usually has easy access to private healthcare and social support in early stages as compared to the upper-middle class. That may be one explanation for low rates of PTSD in this group. It is interesting to note that in our study, gender was not significantly associated with PTSD whereas most research showed that females are more vulnerable to PTSD [57, 59, 60]. This finding is similar to another study of trauma victims of Nepal [22]. One possible explanation for this finding may be better resilience in the population of Nepal, which deserves further exploration. Age of the participant, level of education, place of residence, frequency of trauma and trauma type (earthquake) did not show any significant association with lifetime PTSD, which is similar to the findings from other studies [59, 61, 62].

Participants between 31 and 45 years of age had lower rate of current PTSD than the 18 to 30 years age groups. International studies do not show any significant relationship between age and current PTSD [19, 61]. In Nepal, this age group often has a stable life in terms of work and relationship; thus people may have been able to address their traumatic stress more successfully than other age groups. In our study participants with education above high school were more likely to have current PTSD than those with education less than high school, which is similar to findings from a previously done study in Nepal [22]. In a South African study, however, high education was shown to have a protective role [61]. Yet, other studies do not show any association between PTSD and education [19, 59]. The relationship of current PTSD with education may have other factors influencing it. Factors like lack of employment despite having necessary academic qualifications, the difference in quality of education, cost of education, and burden of educational loans in different countries may influence the relationship of education and current PTSD. Further research addressing these factors and on a larger scale would give more conclusive findings. Exposure to other forms of trauma (other than earthquake) was more likely to have current PTSD than exposure to earthquake. Interpersonal trauma along with intent to cause harm has been seen to have higher risk of causing PTSD [63, 64]. As natural disaster is not an interpersonal type of trauma and does not occur with intent to harm, it may carry a lower risk in comparison to other types of trauma. Similar to our finding, natural disaster has been shown to have significantly less risk of PTSD in comparison to other forms of trauma [41].

Very little research has studied sociodemographic factors associated with depression in the context of trauma [18]. In our study, lifetime depression was significantly lower in the upper class SES in comparison to upper-middle class. This finding is supported by a similar study done in Nepal, in which people with lower SES had more depression [18]. The advantage of better health-care access due to higher SES may be one reason for such an outcome. In a large meta-analysis that studied PTSD and depression in trauma victims, factors like age, gender, place of residence, education, marriage and socioeconomic status did not show any significant correlations [26]. There was also a negative association of upper SES with current depression. One previous study showed significant association only with female gender and the age group 41–50 age group but not with SES [22]. As explained earlier this association may be due to better healthcare accessibility for the upper SES group of participants. Another explanation may also be different types of trauma experienced by the participants.

There are very few studies linking anxiety disorders with sociodemographic variables in trauma patients [18, 65]. Participants from urban areas were found to have a higher chance of having both lifetime and current GAD. This finding is supported by a similar study done in Sudan, which found a significant association between anxiety and an urban place of living [65]. In a study done in the general population of Nepal, anxiety has also been associated with living in urban areas [21]. The rapid influx of villagers into the city, due to decade long civil unrest [66] and earthquake in search of security and better life has caused unplanned urbanization and more hardship in urban areas for this group of people. This might be one possible explanation for the increase of rates of anxiety disorder in people from urban areas. Education above high school was significantly related to current and lifetime GAD. This contrasts a study of trauma victims of Nepal, in which prevalence rate was higher in the illiterate group [18]. One of the major studies done in Nepal on the general population has not explored the relationship of education with GAD and depression [21], citing that since most of the people are illiterate, this variable has less significance. Generally people with higher educational levels in Nepal tend to work more: this increased load of work might predispose them to anxiety symptoms. Further studies exploring this would lead to a clearer picture. People from lower-middle class SES were also found to have a higher chance of having lifetime and current GAD. In a similar study done on trauma-affected populations in Nepal, people with lower SES have been shown to have a higher prevalence of anxiety [18]. However in the general population no environmental factors are associated with GAD [67]. Our findings may be a result of including only trauma patients.

### Strengths and limitations

There are several limitations that suggest caution when interpreting these results. This was a hospital-based study, in which a special group of participants with history of trauma were selected from a psychiatric clinic. Since these participants voluntarily came to seek help for their existing problems, they have a higher probability of having mental illnesses than the general population. Hence, our results cannot be generalized to the general population. With regard to lifetime PTSD, depression and GAD, patients had to recall events from their past, so there is the possibility of recall bias. Furthermore, patients may not have disclosed every sensitive trauma. The duration between trauma exposure and evaluation varied greatly among the participants, which may have led to a variance of prevalence of current PTSD, depression and anxiety symptoms among the participants. In the binary logistic regression models applied here, only lifetime and current anxiety gave significant value in omnibus tests. This might be because the sample size is only 100.

Despite these limitations there are some salient points that make this article relevant. This study is the first hospital-based study in the Asian region in which health outcomes of trauma were evaluated using a semi-structured diagnostic interview by a well-trained psychiatrist. Hence it highlights the current issues related to trauma that have not been well addressed in these special populations.

## Conclusion

The results of our study showed that PTSD and other trauma-related illnesses are highly prevalent among trauma-affected patients coming to the psychiatric clinics of Nepal, though PTSD has historically been underdiagnosed. PTSD carries a bigger burden of disease, as there is high comorbidity with depression and GAD. Therefore, it is advisable for the mental health practitioners in Nepal and in low-income countries to specifically inquire about trauma history and screen for PTSD and related comorbidities in their daily practice. As the sample size in this study was small, further research with a longitudinal design and larger sample is recommended in order to better understand the outcomes of trauma in clinical population.

## Supporting information

S1 Data (SAV)
